# Dr. Jekyll and Mr. Hyde: MAP17’s up-regulation, a crosspoint in cancer and inflammatory diseases

**DOI:** 10.1186/s12943-018-0828-7

**Published:** 2018-04-12

**Authors:** José M. García-Heredia, Amancio Carnero

**Affiliations:** 1 Instituto de Biomedicina de Sevilla, IBIS/Hospital Universitario Virgen del Rocío/ Universidad de Sevilla/Consejo Superior de Investigaciones Científicas, Avda. Manuel Siurot s/n, 41013 Sevilla, Spain; 2 Department of Vegetal Biochemistry and Molecular Biology, University of Seville, Seville, Spain; 3 CIBER de Cáncer, Instituto de Salud Carlos III, Pabellón 11, Madrid, Spain

**Keywords:** MAP17, Cancer, Inflammatory diseases

## Abstract

Inflammation is a common defensive response that is activated after different harmful stimuli. This chronic, or pathological, inflammation is also one of the causes of neoplastic transformation and cancer development. MAP17 is a small protein localized to membranes with a restricted pattern of expression in adult tissues. However, its expression is common in destabilized cells, as it is overexpressed both in inflammatory diseases and in cancer. MAP17 is overexpressed in most, if not all, carcinomas and in many tumors of mesenchymal origin, and correlates with higher grade and poorly differentiated tumors. This overexpression drives deep changes in cell homeostasis including increased oxidative stress, deregulation of signaling pathways and increased growth rates. Importantly, MAP17 is associated in tumors with inflammatory cells infiltration, not only in cancer but in various inflammatory diseases such as Barret’s esophagus, lupus, Crohn’s, psoriasis and COPD. Furthermore, MAP17 also modifies the expression of genes connected to inflammation, showing a clear induction of the inflammatory profile. Since MAP17 appears highly correlated with the infiltration of inflammatory cells in cancer, is MAP17 overexpression an important cellular event connecting tumorigenesis and inflammation?

## Background

Inflammatory response is a common defensive process activated after different harmful stimuli, constituting a highly complex biological mechanism. Through inflammation, the clearance of damaged cells and the removal of pathogens are allowed, initiating both healing and regenerative processes [1, 2]. Macrophages and other inflammatory cells are attracted and activated by signals generated because of the inflammatory process, generating cytokines and proinflammatory chemokines that are released by these cells [3, 4]. As consequence, circulating leukocytes are attracted to the site of inflammation. Once harmful agents have been removed, inflammation also allows the activation of mechanisms of tissue repair [5]. At that way, many cytokines can activate regeneration-activating pathways such as those of YAP, Notch and Stat, all of them involved also in the acquisition of stem cell properties [6–8] . The inflammatory process ends when the activated cells undergo apoptosis in a highly regulated process that finishes after pathogens and cell debris have been phagocytized [9]. Inflammation may turn chronic if the inflammatory cells are not able to eliminate the pathogen. If this happens, a high level of leukocyte infiltration appears in damaged tissues. Many syndromes with an inflammatory component can appear due to this chronic inflammatory condition, like Crohn’s disease, lupus, psoriasis and atherosclerosis [10, 11]. In addition, it has also been connected to Alzheimer’s disease and cardiovascular disease [11, 12].

This chronic, or pathological, inflammation has been also connected to neoplastic transformation and cancer development [13, 14]. At that way, around 25% of the tumors have been highly connected with chronic inflammation derived from an infection, especially stomach cancer [15, 16]. *Helicobacter pylori*, that causes persistent gastritis, increases the risk of developing gastric tumor up to 75% [17]. In addition, the hepatitis B or C viruses increase the risk of developing hepatocellular carcinoma [18]. In addition, chronic inflammation in organs like pancreas or prostate is commonly followed by the appearance of tumors [19, 20]. Crohn’s disease increases the risk of developing colorectal cancer by up to ten-fold [11]. As consequence, all these tumors, with an important inflammatory component in its origin, are characterized by the presence of immune cells and mediators of inflammation, being able leukocytes to constitute up to 50% of the total tumoral mass [21, 22]. Cancer cells and macrophages interaction stimulates the latter to produce proinflammatory cytokines, like IL-8, attracting as consequence additional inflammatory cells [4]. The subsequent inflammatory microenvironment has been accepted as an essential component of all tumors [21, 23]. Recent efforts have been dedicated to understand the tumor-elicited inflammation, an inflammatory reaction necessary for tumor development and detected in many solid malignancies [24–28].

*MAP17*, also known as *PDZK1IP1*, *DD96* or *SPAP* [29–31], was identified as an upregulated gene in the malignant epithelial cells of renal cell carcinomas [29]. It was also identified in a genome-wide retroviral cDNA screen designed to search for genes conferring selective advantage to cells during tumorigenesis [32]. MAP17 expression is restricted to specific epithelial cell populations associated with the apical brush border, such as the proximal tubular cells of the normal kidney, however, it has been shown to be a gene commonly upregulated in tumors, being overexpressed in more than 50% of the advanced tumors or metastases analyzed [31]. MAP17 is overexpressed in most human carcinomas and in other non-epithelial neoplasias, such as glioblastomas or lymphomas [33, 34]. In addition, MAP17 overexpression is a common feature of tumors and is associated with tumor progression, being correlated with two of the most important events leading to a malignant phenotype: cellular immortalization and transformation [31, 35, 36]. Furthermore, we have recently shown that high MAP17 expression is not restricted to cancer [37].

By its PDZ-binding domain, MAP17 acts as a carrier from the Golgi to the cell membrane increasing protein membrane loading and inducing the attraction of inflammatory cells. Furthermore, MAP17 also modifies the expression of genes connected to inflammation, showing a clear induction of the inflammatory profile. Thus, MAP17 expression regulates the expression of inflammation-related genes, through induction of genes like NFAT2 and IL-6. Therefore, the expression of MAP17 triggers chronic inflammation not only in cancer but in various inflammatory diseases such as Barret’s esophagus, lupus, Crohn’s, psoriasis and COPD. Since MAP17 appears highly correlated with the infiltration of inflammatory cells in cancer, does MAP17 expression triggers chronic inflammation in tumors? In the present manuscript we will review the data about this gene and its relevance in tumorigenesis and chronic inflammation, and the essential role of MAP17 in both.

## Main text

### MAP17 overexpression increases tumorigenic potential

Due to the increased expression of MAP17 found in advanced tumors and metastases, it is important to explore the consequences of MAP17 expression in human cells. In this way, most of the research done so far has been focused on what occurs in cells with increased MAP17 expression. Likewise, initial experiments using forced expression of MAP17 in non-tumoral immortalized human mammary epithelial cells (HMECs) showed a decrease in both cell proliferation and tumor growth, and cells appeared to take on a senescent morphology [34, 38]. This advanced stage of differentiation, or senescence, has been shown to be related to MAP17-induced increases in ROS. This effect seems to be related to p38α phosphorylation because shRNA against p38α was able to overcome the MAP17-induced senescence and allowed for MAP17-dependent tumorigenesis of immortalized HMECs [39].

Most of the research done so far concerning the role of MAP17 in cancer has shown that MAP17 works as an oncogene, increasing tumorigenicity when it is overexpressed [31, 40–43]. In addition, MAP17 levels are correlated with tumoral progression, being higher in late-stage or metastatic tumors than in benign tumors or normal tissues. MAP17 overexpression has been found in the advanced stages of ovarian, cervical, laryngeal and prostate cancer [31]. Likewise, a high percentage of advanced tumors (50–90%) exhibit high levels of MAP17, and this expression is correlated with an increase in cell dedifferentiation [35, 36, 39].

Increased MAP17 levels have a marked effect on tumor progression [31, 39–41]. Tumor cells lines with low MAP17 always exhibit an increase of tumorigenic properties due to MAP17 ectopic overexpression. Assays with A375 melanoma cells showed that cells overexpressing MAP17 grew faster than control cells and thus had a proliferative advantage [40]. Ectopic MAP17 expression induced an increase in the proportion of holoclones, which can be related to an increase in stem cell-like properties, as it has been previously described [44–49]. In addition, soft agar and tumorsphere assays also showed an increase in the number and size of colonies formed compared to parental cells [39, 40]. Additionally, MAP17 overexpression in cancer cells reduced the percentage of apoptotic cells and induced an increased growth ratio in mouse tumors [39, 40].

On the other hand, MAP17 downregulation by shRNA, both in MAP17-overexpressing cells, for example, in Calu3 or MDA-MB-431 cells, that naturally express high levels of MAP17, reduced tumorigenic properties [39, 41]. Similarly, a decrease in colony size and a reduced number of colonies were observed due to MAP17 downregulation, indicating that MAP17 expression is required to maintain increased tumorigenic properties. Other experiments using siRNA against MAP17 in thyroid cancer cell lines additionally impaired cell migration and invasion [50]. The downregulation of MAP17 also reduced the number and size of tumorspheres and of holoclones, indicating the MAP17-induced regulation of the cancer stem cell pool [51].

Therefore, the overexpression of MAP17 increases the cancer stem cell-like pool of tumor cells, independently of the tumor origin of the cells, while the downregulation of the protein in cells that endogenously expresses it, triggers the reduction of this cancer stem cell pool [51]. This feature has been related to the activation of the Notch pathway as we will explore later. Importantly, this feature may explain the increase in the tumorigenic properties and the resistance to apoptosis observed in tumor cells.

### MAP17 and inflammation

Downregulation of SLC34A2, a Na^+^-dependent phosphate transporter correlated with MAP17 expression [52], has been associated with the initiation and progression of lung adenocarcinoma through AKT/PI3K activation [53]. In this study, MAP17 appeared to be correlated with complement genes (*C3*, *C4b*, *C5*) and complement-associated genes (*FGA*, *FGB*, *FGG*), suggesting possible activation of the alternative complement pathway, an usual event in cancer cells [54]. The complement system is associated with both inflammatory diseases and cancer [55], showing that MAP17 could have an important role not only in cancer but also in inflammation. In this way, MAP17 was found to be significantly overexpressed in patients with Crohn’s disease and ulcerative colitis compared to its expression in normal patients and caused significant changes in six additional genes (*CXCL1*, *MMP7*, *SLC6A14*, *SLC26A2*, *REG4*, *VNN1*) [56].

Although both studies pointed to a possible role of MAP17 in inflammation, there were no data on what that role could be. However, two studies have shown that MAP17 can induce an inflammatory phenotype, similar to the phenotype that occurs when MAP17 increases tumorigenic properties. The first study established a relationship between MAP17 and filaggrin, a cornified envelope-associated protein usually downregulated in inflammatory skin diseases such as atopic dermatitis [57]. In a meta-analysis of microarray studies of four different dermatological diseases (dermatitis, acne, nickel allergy and psoriasis), MAP17 expression was correlated with a profile of cytokines (IFN-γ, IL-4, IL-6, IL-17A, IL-17F, and IL-22) that induced transcriptional downregulation of filaggrin [58]. Indeed, the overexpression of the C-terminal, cytoplasmic fragment of MAP17, from amino acid 59 to 114, in keratinocytes decreased the expression of filaggrin [58]. Additionally, keratinocyte treatment with IFN-γ, IL-4, IL-6, IL-17A or IL-22 induced MAP17 expression, showing its connection with the inflammatory response [59]. In inflammatory diseases such as psoriasis or Crohn’s disease, cytokines are secreted by T-helper cells [60, 61], and it is hypothesized that MAP17 upregulation is a secondary effect of this increased cytokine secretion. Like for epidermal diseases, the higher amount of T-helper cells in tumors suggest that MAP17 upregulation could also be the result of the upregulation of several cytokines [58]. Interestingly, the gene for PDZK1, a known protein that interacts with MAP17 [62], is localized within the atopic dermatitis-linked region on human chromosome 1q21, such as the filaggrin gene [58].

In line with these data, in a recent study, we showed that MAP17 directly regulates the expression of genes related with the inflammatory response, such as HLAs, IL-6 and NFAT2 [63–65]. Therefore, cells with high MAP17 levels also exhibit increased cytokine (i.e., IL-6) secretion, which induces differentiation of monocytes to dendritic cells [37]. As such, an increased MAP17 level is an important event both in cancer and in inflammatory diseases, which makes it possible to design common strategies that can treat both diseases similarly in cases with MAP17 overexpression.

### *MAP17* gene

**Fig. 1:**
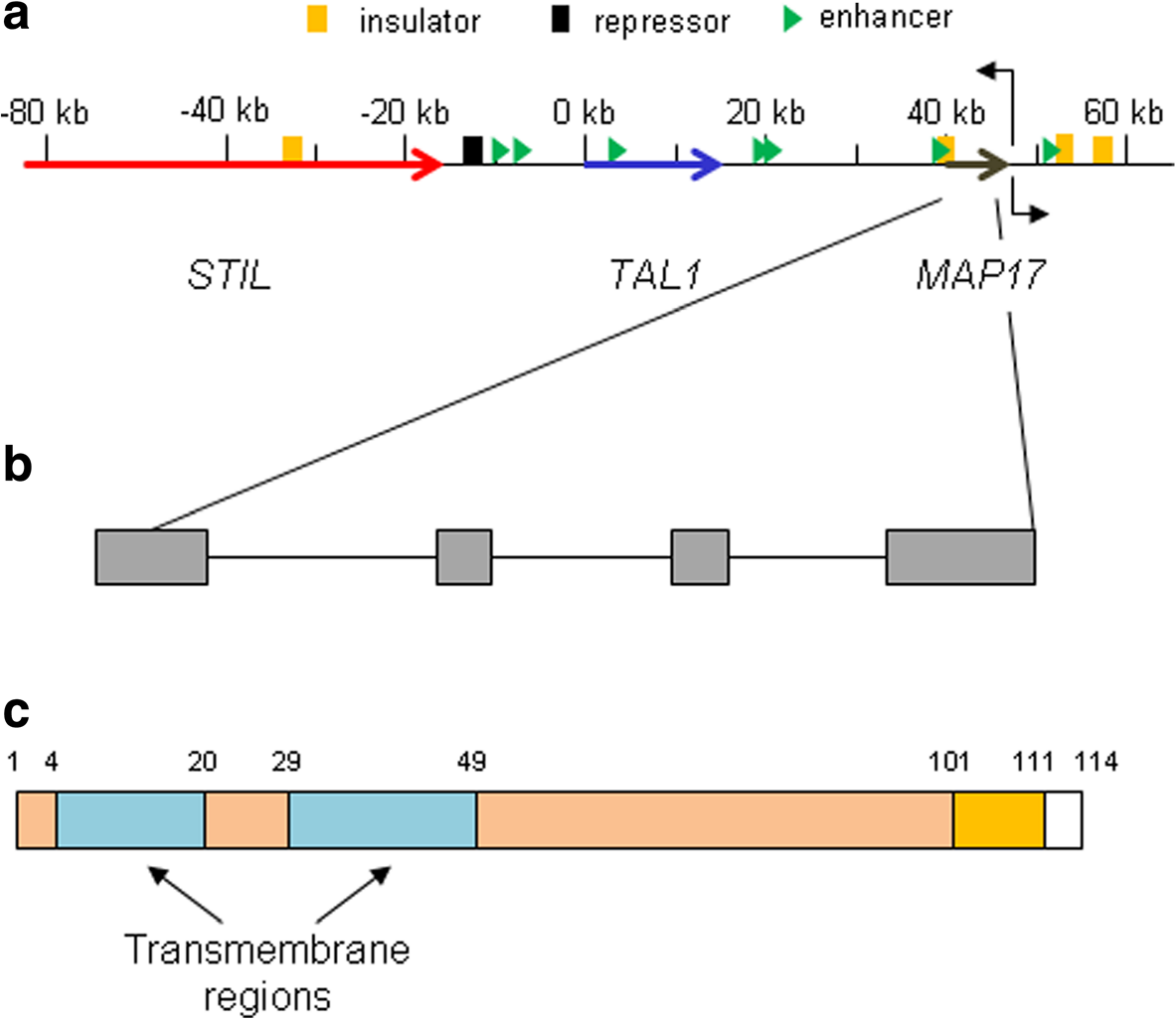
**a** Schematic representation of the SCL/TAL1 locus in chromosome 1p33, showing the positions of *STIL*, *TAL1* and *MAP17* genes, as well as the regulatory regions. **b** MAP17 gene, composed by 4 different exons. **c** MAP17 protein, showing the transmembrane domains, the PDZ-binding domain (white), and the interaction region with NUMB (orange)

Human MAP17 is encoded on chromosome 1p33 in the SCL/TAL1 locus, where TAL1, a basic HLH protein essential in the production of the hematopoietic lineages, and the 3′ sequence of STIL, are localized (Fig. 1a). In addition, other genes coding for members of the cytochrome P450 family (*CYP4B1*, *CYP4A11*), *MCPH7*, *CMPK1* and members of the forkhead family (*FOXE3*, *FOXD2*) are also localized close to *MAP17* gene [35]. *STIL*, *TAL1* and *MAP17* genes constitute a genomic region of approximately 88 kb with multiple regulatory elements, both enhancers and repressors [66]. *MAP17* shares some regulatory elements with *TAL1*, and the expression of both genes is correlated in all hematopoietic cell types where *TAL1* is expressed. In fact, *MAP17* is immediately positioned at the 3′ end of the hematopoietic master regulator *TAL1* [67, 68]. Deletion of this enhancer, which is evolutionary conserved, reduced both *TAL1* and *MAP17* expression [69, 70]. In addition, *MAP17* upregulation has been detected during megakaryocyte differentiation from CD34^+^ hematopoietic progenitor cells [71]. The SCL/TAL1 locus is also regulated through histone acetylation, and one of these acetylation sites is localized over the *MAP17* promoter. This modification, found in renal tubule cells with high MAP17 levels, could be related with active transcription of this gene [67]. Two non-coding RNAs, named *ncRNA-a3* and *ncRNA-a4*, have been detected close to *MAP17* gene in the SCL/TAL1 locus, although a possible effect on *MAP17* expression remains to be characterized [72].

However, it has also been shown through bioinformatics analysis that *TAL1* and *MAP17*, although correlated, do not share strong network connections during erythroid differentiation, suggesting they may have independent functions [73]. In fact, MAP17 expression in tumors is not correlated with TAL1 expression. While MAP17 levels are typically increased in tumor samples compared to levels in non-tumoral samples, no changes in the expression of *TAL1* have been observed, showing that, at least in cancer cells, *MAP17* transcription is independent [31].

The *MAP17* gene itself is divided into four different exons that together generate a 600 bp transcript (Fig. 1b). Up to now, only two different mutations have been described affecting this gene. One of them, C403T, was identified in malignant pleural mesothelioma tumors and causes an amino acid substitution, T79I, in the C-terminal cytoplasmic region [74]. The second identified mutation, C176 + 1G > A, is in the boundary between exon 2 and intron 2 and causes a shift of the reading frame in such a way that activation of SGLT2, a protein related with MAP17, is no longer possible [75]. This mutation turned MAP17 into a non-functional protein and caused loss of the phenotype typically observed when the wild-type gene is expressed. However, MAP17 expression in tumors usually increases due to progressive demethylation and/or oncogenic activation of the promoter [29, 31]. Both effects cause an increase in protein expression, mostly through mRNA amplification [31, 34]. It has been shown that *MAP17* is differentially upregulated in distant metastases [30].

### MAP17 protein

MAP17 is a small, non-glycosylated protein of approximately 17 kDa usually localized to the plasma membrane of cells and is associated with areas of cell-cell contact [34]. Although its structure is not known, it has a double hydrophobic region that allows it to function as an anchored membrane protein [38, 76]. It seems to form homodimers of 24 kDa, probably through a disulfide bond between intracellular Cys55 residues [77]. No functions have been reported for the N-terminal domain, with the exception of a potential signal peptide for its localization [29]. MAP17 functionality is localized to its C-terminal sequence, which contains a PDZ-binding domain composed of the last four amino acids (STPM) that allows its interaction with several PDZ domain-containing proteins (Fig. 1c) [35, 38, 76, 78]. In addition, the MAP17 PDZ-binding domain is essential to increase cell growth rate, clonability, growth in soft agar and tumorspheres size and/or number [40, 41].

Although the function(s) of MAP17 have not been fully determined yet, it has been postulated that its primary role is to act as a cargo protein, allowing the movement of proteins from the Golgi apparatus to the plasma membrane [32, 41, 76, 77]. The PDZ-binding domain of the MAP17 C-terminal region allows for the configuration of a complex network of protein interactions that will be discussed below.

In normal human cells, its expression is restricted to specific epithelial cells from the kidney (proximal tubular cells), epidermal keratinocytes and the spermatids of the seminiferous tubules [34, 38]. In addition, it has also been detected in the kidney, lung and liver of adult mouse tissues and in the proximal tubules of the kidney cortex and the spermatids of the seminiferous tubules of rats [77]. It has also been shown to be correlated with PDZK1 [79].

#### MAP17 and PDZK1: An interaction that allows for the construction of a variable complex

One of the first proteins described to interact with MAP17 was PDZK1 [62, 78, 79]. MAP17-PDZK1 interaction occurs between the STPM sequence of MAP17 and one of the PDZ-binding domains of PDZK1. In this way, MAP17 interaction with PDZK1 and other proteins, such as NaPiIIa and NHE3, is disrupted by the deletion of the STPM sequence from MAP17 [39, 76, 79]. All published data about MAP17 in non-tumor cells point to its role as a membrane protein anchoring the PDZK1 protein. Similarly, it has been described that MAP17 overexpression in a transgenic mouse model caused PDZK1-induced liver deficiency, suggesting that MAP17 could act as an endogenous regulator of PDZK1 turnover in situations of increased degradation of PDZK1 and higher HDL plasma levels [79]. MAP17 together with PDZK1 and MRP2 (multidrug resistance-associated protein 2, also called cMOAT or ABCC2) may constitute a heteromultimeric protein complex [62]. These proteins together may have an important role in multidrug resistance because proteins with PDZ domains, such as PDZK1, are involved in recruiting membrane-associated proteins that are involved in many different signaling pathways [35].

PDZK1 belongs to the NHERF (sodium hydrogen exchange regulatory factor) family, which comprises PDZ domain-containing proteins with important roles in cell function regulation. This family is constituted by four members, NHERF1, NHERF2, PDZK1 (NHERF3) and IKEPP (NHERF4), with similar homology domains [80], although NHERF1 and NHERF2 have 2 PDZ domains, while PDZK1 and IKEPP have 4 PDZ domains [81]. Proteins of this family are mostly expressed on the apical side of polarized epithelial cells, mainly from the kidney, small intestine and liver [82, 83]. These proteins can regulate the functional activity and expression of cell surface transporters, most of which are part of the ABC family [81]. Other important elements, such as signaling proteins, hormone receptors and cytoskeleton structural elements, also interact with NHERF proteins [80, 84].

Regarding individual members of NHERF family, it has been shown that NHERF1 and NHERF2 are important in inhibiting the Na^+^-H^+^ exchange isoform 3 (NHE3). PDZK1 is a scaffolding protein that allows for the assembly of several proteins into functional complexes and is a critical regulator of intracellular signaling in response to specific stimuli [81]. PDZK1 and NHERF1 can form hetero-oligomers that likely allow for the formation of an entire network of PDZ adapter proteins beneath the plasma membrane [85]. Similarly, PDZK1 regulates the solute carriers SLC9A3 (sodium/hydrogen exchanger, NHE3) [86], SLC15A1 (oligopeptide transporter, PEPT1) and SLC22A5 (carnitine/organic cation transporter, OCTN2) in the small intestine [87] and regulates the cystic fibrosis transmembrane conductance regulator (CFTR) [88] and the anion exchangers of the SLC26A family, leading to PDZK1 stabilization. The binding of CFTR to DRA and PAT1 results in activation of the Cl^−^/HCO_3_^−^ exchanger. In addition, PDZK1 also interacts with AKAP10, FARP2, sodium-hydrogen antiporter 3 regulator 1, SLC22A12, SLK, SLC22A4, CLCN3, and SLC34A3 [89]. In this way, PDZK1 can regulate the activity of cation and anion transporters, modifying cell membrane properties according to cellular needs.

The described interaction of MAP17 with PDZK1 allows for its function as an atypical anchoring site for PDZK1 and other NHERF proteins, and allows for MAP17 interaction with the NaPi-IIa/PDZK1 protein complex in renal proximal tubular cells [78]. In this way, MAP17 overexpression in opossum kidney cells, together with PDZK1 and IKEPP, allows for internalization of NaPiIIa from the apical membrane into the trans-Golgi network [76, 78]. The complex composed of MAP17, NHERF1 and PDZK1 may alter the membrane localization of pumps and transporters and thus deregulate the intracellular and extracellular cation/anion equilibrium [35]. In spite of the described PDZK1-MAP17 interaction, PDZK1 downregulation had no effect on MAP17 expression nor in its cellular distribution [90]. These results suggest that PDZK1 is not required for the membrane localization of MAP17.

#### MAP17 influences glucose/mannose uptake by SGLT1/2 regulation

MAP17 overexpression has also been shown to stimulate specific Na^+^-dependent transport of mannose and glucose in *Xenopus* oocytes and mammary cells [32, 77]. In this way, it has been shown that this protein modifies the activity of SGLT1 and SGLT2 (Na^+^-dependent glucose transporters 1 and 2) [36, 75]. These transporters are mediators of apical glucose uptake in intestinal cells. When coupled to the Na^+^/K^+^ ATPase to provide the necessary energy, GLUT1, a glucose transporter, facilitates the diffusion of intracellular glucose from the basolateral membrane to the bloodstream [91]. In this way, glucose is efficiently transported from the lumen of the small intestine to the blood.

Although SGLT1 and SGLT2 do not have PDZ domains nor PDZ-binding domains, their activities are modulated by MAP17/PDZK1 expression, suggesting that other factors may be involved. MAP17 has been identified as a factor stimulating the uptake of α-methyl-glucose, a non-metabolizable glucose analog, by SGLT2, increasing its ability to transport glucose [75]. In fact, MARDI protein, which shares a common amino acid sequence with MAP17 within its two transmembrane domains, can also stimulate SGLT2 [75]. Given that the addition of glucose activates PDZK1-mediated Na^+^/HCO_3_^−^ cotransport [92, 93] and that the activity of SGLT2 is potentiated by MAP17 [75], SGLT2, MAP17 and PDZK1 could be part of a signaling complex. Although SGLT1 activity is not directly dependent of MAP17, both proteins appear to be linked in cervical cancers. In line with this, glucose uptake was increased an average of 4-fold in cells overexpressing MAP17, an effect which could be inhibited by treatment with the SGLT inhibitor phloridzin [36]. In addition, a significant positive correlation between SGLT1 and MAP17 was found in different cancers [36].

PDZK1 and SGLT1/2 appear to be connected given the described function of PDZK1 as a platform for ion transporters and the connection of SGLT1/2 with the transport of glucose in a sodium-dependent manner [36, 75]. In addition, the ability of MAP17 to modify the localization of ion transporters identifies it as a regulatory protein for ion and glucose transport, which it accomplishes by competitively binding to PDZ-binding domains to alter the stoichiometry of the transporter-PDZ proteins [77, 94].

### MAP17 overexpression modifies cell signaling pathways

Changes in the tumorigenic properties of cells overexpressing MAP17 suggest that these cells likely have modifications in their signaling pathways. Research done so far has shown that MAP17 overexpression causes a pleiotropic effect, modifying several signaling pathways. Here, we describe the effects on different signaling pathways induced by MAP17 overexpression.

#### MAP17 overexpression allows for activation of the notch pathway

Aberrant activation of the Notch signaling pathway has been reported both in cancer cells and in tumors and is correlated with increased stem-like properties in tumor cells and with cancer metastasis [41, 95, 96]. Notch protein, which is localized to the plasma membrane in its inactive state, interacts with ligand receptors on neighboring cells and is then proteolytically processed by γ-secretase. As a consequence of this processing, the Notch intracellular domain (NICD) is released into the cytoplasm, resulting in the active form of Notch. Translocation of the NICD to the nucleus allows for the transcription of Notch target genes, including genes from the *HES* and *HEY* families [97, 98]. The Notch pathway is negatively regulated by NUMB, which interacts with the NICD in such a way to interfere with its nuclear translocation and to allow for the ubiquitination of the NICD prior to its proteasomal degradation [99–101]. As such, it has been shown that lower NUMB levels are correlated with increased Notch signaling and increased tumorigenic properties [102, 103]. In addition, tumors with decreased NUMB levels are correlated with a worse prognosis and a more dedifferentiated state [103–105], sharing features with tumors expressing MAP17.

MAP17 overexpression activates the Notch pathway due to the direct interaction between MAP17 and NUMB in tumor cells [41]. Because this interaction is mediated by the last 13 amino acids of MAP17, deletion of this region (tMAP17, truncated MAP17) blocked the interaction of MAP17 with NUMB and resulted in a loss of MAP17-induced tumor-promoting properties. In this way, cells overexpressing tMAP17 behaved such as control cells. Consistent with this, both MAP17 overexpression and NUMB downregulation by specific shRNA caused similar effects, with an increase in nuclear NICD and an increase in the expression of Notch-related genes, including *HES1* and *HES5* [41, 106]. In addition, stem-cell related genes and cell surface markers, including *OCT4*, *NANOG*, *SOX9*, *KLF4*, CD44 and CD133, were upregulated in both conditions, suggesting an increase in stem-cell properties in cells, which was confirmed by tumorspheres and clonal growth assays. In addition, analysis of human cancer datasets confirmed that MAP17 correlated with genes from Notch pathway.

These experiments show that one of the main effects driving the oncogenic effect of MAP17 is its interaction with NUMB, which allows for aberrant activation of the Notch pathway. However, MAP17 overexpression is also related with other pro-oncogenic effects.

#### MAP17 overexpression inhibits NFκB pathway

In addition to its effect on Notch signaling, it has been shown that MAP17 overexpression reduces NFκB activation and cell autophagy [32]. NFκB is known to activate antiapoptotic genes and promote cell survival, and the NFκB pathway has been described as an important survival mechanism in tumor cells [107]. MAP17 prevents the cytoprotective activation of NFκB and cell autophagy induced by bortezomib (velcade, PS-341) [42, 43]. Cells with higher MAP17 expression levels showed decreased phosphorylation of NFκB, with a reduced nuclear accumulation of NFκB-p65, lower IκBα levels, and lower levels of autophagy, suggesting the high relevance of these two pathways in MAP17-induced drug sensitivity [42, 43]. Cells overexpressing MAP17 also showed decreased ERK1/2 phosphorylation levels at lower bortezomib concentrations [43]. Bortezomib also induces autophagy, a process characterized by the intracellular formation of the autophagosome, a double-membrane vesicle in which cellular components are digested and recycled [108, 109]. Cells overexpressing MAP17 reduced bortezomib-induced autophagy [43].

Tumor necrosis factor (TNF) is one of the most important cytokines with a wide range of roles (from DNA fragmentation to NFκB phosphorylation), being bypassed by MAP17 overexpression. In this way, MAP17 blocks TNF-induced growth arrest by inhibiting p21 activation and pRB dephosphorylation. TNF inhibition due to MAP17 expression is specific, because MAP17 does not alter the response of other cytokines, such as IFN-γ [32].

Fibroblasts expressing c-Myc enter apoptosis under low serum conditions [110]. However, MAP17 overexpression reduced the percentage of cells expressing c-Myc that entered apoptosis, with a reduction in caspase-3 activity of up to 60% [33]. The protective effect exerted by MAP17 is due to the activation of the AKT/PI3K pathway because cells overexpressing MAP17 were insensitive to serum starvation and showed no AKT dephosphorylation, which promoted cell survival [33].

### MAP17 overexpression increases reactive oxygen species production

Reactive oxygen species (ROS), such as superoxide (O_2_^−^), hydroxyl radicals (OH^−^) and hydrogen peroxide (H_2_O_2_), are considered to be by-products of the mitochondrial electron transport chain [111]. Under physiological conditions, antioxidant enzymes, such as superoxide dismutase, catalase, and others, exist in a delicate balance with these oxidative inputs, protecting cells from the oxidative stress induced by ROS [112]. It has been described that moderate to high levels of ROS promote cell proliferation, survival and migration [113, 114]. However, altered ROS levels promote various pathological conditions, including cancer. ROS-induced oncogenic effects include changes in gene expression regulation, increased mutagenic rates and genomic instability [39, 114]. After oncogenic transformation, cells rapidly activate a stress response as a protective measure to overcome oncogene-induced cell death and senescence [115].

Although ROS increase is a typical feature of cancer cells and is partially responsible of the enhanced malignant properties of tumor cells, it is also a potent proapoptotic stimulus [42, 116]. ROS generation in tumors, and the subsequent oxidative stress, actually occurs at sublethal levels [117], making it easier to reach levels causing cell death [35, 118]. Therefore, although many cancer cells can tolerate limited doses of ROS, excessive intracellular ROS accumulation overruns detoxification enzymes, and apoptosis is initiated [119]. Thus, patients with tumors expressing high ROS levels could benefit from therapies increasing oxidative stress. However, antioxidants and low ROS levels that induce antioxidant defenses both appear to benefit tumor growth and could enhance anticancer therapy resistance [120]. As such, understanding the duality of ROS as cytotoxic molecules and key mediators in signaling cascades may provide novel opportunities to improve cancer therapeutic interventions [121].

Mitochondrial defects in oxidative phosphorylation (OXPHOS), which are typical of tumor cells, cause reduced ATP production, with most of the increases in ROS being due to the electron transport chain inhibition because of OXPHOS defects [122]. Lower ATP levels force tumor cells to have a high rate of glycolysis, supported by an increase in glucose uptake usually 20–30-fold higher in tumor than in normal cells [123]. This enhancement in glucose uptake occurs via GLUT1 and SGLT1 activation [123]. As a consequence of increased glycolysis, tumor cells accumulate lactate, which affects them negatively through changes in intracellular pH [124, 125]. This effect forces the cells to regulate their intracellular pH to survive, enhancing the expression of membrane-localized transporters and exchangers. Some of these membrane transporters are regulated by MAP17 levels and are normally localized by NHERF proteins binding [35, 126, 127].

Although MAP17 has no enzymatic activity, its overexpression causes an increase of 30–40% in ROS generation levels, compared to control cells [35, 39, 40]. In fact, MAP17 overexpression alters the mRNA levels of genes regulating oxidative stress [36, 43]. Because MAP17 effects SGLT1/2 to enhance glucose and mannose uptake, glycolysis is activated, and ROS levels are increased as a by-product [36, 75, 77]. In addition, changes in the MAP17-PDZK1 interaction could also alter the intracellular and extracellular ion balance, thus modifying the intracellular redox balance [35, 40]. As such, the increased ROS levels induced by MAP17 overexpression are partially responsible for the enhanced tumorigenic properties of cancer cells overexpressing MAP17 and for the previously described modified cellular pathways [35, 40]. It is interesting to note that MAP17 without its PDZ-binding domain is unable to increase tumorigenic properties and unable to increase ROS production [40].

High ROS levels activates the AKT/PI3K pathway by direct oxidation and inactivation of PTEN and other AKT phosphatases, thus maintaining AKT activation even in the absence of a PI3K signal [33]. PTEN is responsible for AKT dephosphorylation and is inactivated by oxidation under ROS stress [128, 129]. The levels of oxidized PTEN in MAP17-overexpressing cells under serum deprivation conditions that potentiate ROS production was up to 10-fold higher than in control cells, indicating that the AKT/PI3K pathway remains activated [33]. Under the same serum deprivation conditions, treatment of MAP17-overexpressing cells with antioxidants prevented the activation of AKT and restored the level of apoptosis [33]. Inactivation of p38α looks to be an essential step due to its role as a sensor for ROS levels, and these levels can simultaneously act as a switch for the tumorigenic and suppressive actions of p38α [130–132]. As it was stated above, active, phosphorylated p38α in non-tumor cells blocks the tumorigenic transformation induced by MAP17. Both the inactivation of p38α and its downregulation by shRNA caused faster growth and higher colony numbers in cells overexpressing MAP17 [39]. AKT pathway activation by MAP17 expression may lead to diminished p38α phosphorylation, probably through the activation of kinases upstream of p38, such as ASK1 or MEKK3. Most likely, ROS production due to MAP17 overexpression results in the oxidation of certain cysteine residues of thioredoxin, which induces the dissociation of p38α from ASK1, a kinase involved in p38α activation. Thus, prior p38α inactivation is necessary for the higher ROS levels and increased tumorigenic properties caused by MAP17. This makes cells with no p38α more susceptible to MAP17 oncogenic alteration [39].

It has been shown that cells with high MAP17 levels are more sensitive to treatments that increase ROS production, likely because the increased ROS can tip the balance towards apoptosis. As such, the combination of MAP17 overexpression with antioxidants in rat fibroblasts overexpressing c-Myc reduced the survival of MAP17-expressing clones, suggesting that ROS generation acts as a mediator of MAP17-induced survival through PI3K/AKT signaling [33]. In cells overexpressing MAP17, antioxidants reduced the oncogenic properties of MAP17, decreasing both the number and size of colonies of MAP17-expressing cells [40]. In addition, treatment of non-tumor cells overexpressing MAP17 with antioxidants reduced the occurrence of senescent cells [39]. These findings showcase MAP17 expression as an interesting biomarker for predicting prognosis and treatment design.

### MAP17 as a biomarker for prognosis

**Fig. 2:**
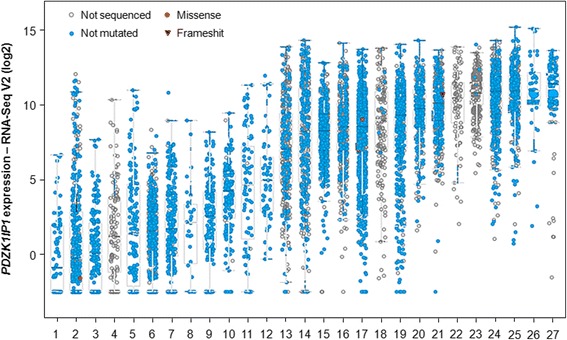
Levels of *MAP17* (*PDZK1IP1*) mRNA expression in different tumor types. Data obtained from cBioPortal. 1: Uveal melanoma; 2: Melanoma; 3: PCPG; 4: Thymoma; 5: Testicular germ cell; 6: Glioma; 7: Sarcoma; 8: DLBC; 9: AML; 10: GBM; 11: ACC; 12: Uterine CS; 13: Bladder; 14: Liver; 15: Prostate; 16: Lung squamous; 17: Breast; 18: Uterine; 19: Thyroid; 20: Ovarian; 21: Lung adenocarcinoma; 22; Mesotheolioma; 23: Colorectal; 24: Head & neck; 25: Cervical; 26: Cholangiocarcinoma; 27: Pancreas

Although the expression of MAP17 can be detected in all tumor types, epithelial tumors showed a higher percentage of tumors with high MAP17 expression levels (Fig. 2). In some cases, as in pancreatic adenocarcinoma, the high percentage of tumors with MAP17 overexpression could be due to the late stage identification and analysis of the samples. Therefore, MAP17 is overexpressed in a great variety of human carcinomas. Immunohistochemical analysis of MAP17 during prostate, breast, and ovarian carcinoma progression shows that overexpression of the protein strongly correlates with tumoral progression [31, 36, 39]. Many tumor samples and tumor cells also express MAP17, and its expression does not correlate with expression of SCL, the neighbor gene reported to be co-expressed in some hematopoietic cell lines [31]. SCL is also not expressed in most MAP17-positive tumors, indicating the independent transcription of MAP17, at least in carcinomas [31]. We cloned the 5′ sequence of MAP17 and determined the minimal promoter sequence necessary to produce independent activation of MAP17 [31]. Moreover, we have found that the MAP17 promoter is activated by oncogenes [29, 31]. Taken together, these findings suggest that there is independent activation of the MAP17 promoter that can be driven by oncogenes, which might explain the common overexpression of MAP17 in human carcinomas. However, MAP17 is strongly demethylated during tumor growth and might contribute to tumor growth in thyroid cancer [133].

We analyzed the expression of MAP17 in sarcomas and its relationship with clinicopathological features [42]. We found that the levels of MAP17 were related to clinical features and poor survival in a cohort of patients with different sarcoma types, and MAP17 expression was not restricted to any specific tumor subtype [42]. MAP17 expression is associated with poor overall survival and worse disease-free survival. However, MAP17 and SGLT1 were expressed in approximately 70% and 50% of cervical tumors of different types, respectively, but they were not expressed in adenoma tumors [36]. Furthermore, there was a significant correlation between MAP17 and SGLT1 expression levels. High levels of either MAP17 or SGLT1 correlated with improved patient survival after treatment [36]. However, the patients with high levels of both MAP17 and SGLT1 survived through the end of the study. Therefore, the combination of high MAP17 and SGLT1 levels is a marker for good prognosis in patients with cervical tumors after cisplatin plus radiotherapy treatment [36]. In larynx cancer, MAP17 expression is associated with better overall survival and laryngoesophageal dysfunction-free survival. Locoregional control in patients with high MAP17 showed better outcomes than those with low MAP17 [134]. In addition, a positive correlation was observed between MAP17 expression and SGLT1, and the combination of high levels of MAP17 and SGLT1 also led to increased overall survival [31, 134]. These findings suggest that MAP17, alone or in combination with SGLT1, may become a novel predictive biomarker for laryngeal carcinoma, another tumor with similar therapeutic options, and for response to platins and radiotherapy. In a follow-up study, patients with larynx cancer were evaluated to determine whether γH2AX phosphorylation (pH2AX), a component of the histone octamer in nucleosomes which is phosphorylated upon DNA damage, alone or in combination with the membrane protein MAP17 could be used as a prognostic biomarker [135]. The authors found that the dose of cisplatin but not the length of radiotherapy influenced LDS. High-pH2AX expression was associated with prolonged LDS while MAP17 correlated with overall survival (OS). High-MAP17 and high-pH2AX combined analysis showed improved LDS (with 61.35 months vs 32.2 months) and OS (with 66.6 months vs 39.8 months). Furthermore, the subgroup of patients with high-pH2AX and an optimal dose of cisplatin was also associated with OS (72 months vs 38.6 months) and LDS (66.9 months vs 27 months). These findings suggest that pH2AX alone or in combination with MAP17 may become a novel and valuable prognostic biomarker for patients with laryngeal carcinoma treated with preservation approaches.

### MAP17 guided therapeutic intervention

As we stated above, MAP17 expression correlates with higher tumor grade and poorer differentiation. This can be used as a tool to treat tumors with a poor prognosis [36, 121, 134].

To explore the role of MAP17 as a predictive biomarker of response to antitumor treatments, we performed a search for MAP17 partners to identify a functional relationship between MAP17 and a cellular process suitable for targeting. In this search, we identified 184 proteins, most of which belong to the proteasomal degradation pathway. Because the inhibition of proteasomal function has been described as a suitable antitumor strategy for some cancers, we tested whether interfering with proteasomal function might constitute a valuable therapeutic strategy in MAP17-expressing cells. Bortezomib inhibits 20S proteasome function and possesses potent antitumor activity both in vitro and in xenograft models of different tumors [136–138]. Bortezomib induces the unfolded protein response (UPR), which is activated in response to alterations in the ER physiological environment, and induces ROS production [139, 140]. We found that cells overexpressing MAP17 are more sensitive to bortezomib, and patients with higher MAP17 mRNA levels respond better to this therapy and exhibit prolonged survival [43]. We also showed that MAP17 determines the bortezomib sensitivity by inhibiting the cytoprotective effects related to bortezomib-induced NFκB nuclear translocation and autophagy. Furthermore, inhibition of oxidative stress abolishes the sensitivity to bortezomib induced by MAP17 [43]. In tumors with high MAP17 levels, this protein could functionally act as an autophagy inhibitor, removing the need for synergistic treatment with an autophagy inhibitor in cancer treatment. This has a clear beneficial effect: reduced toxicity in patients due to the restricted expression of MAP17 in normal tissues. As such, no other secondary effects are expected to arise due to treatment [43].

Therefore, high levels of MAP17 could be used as a prognostic marker to predict the response of patients with diseases that can be clinically treated with bortezomib. Additionally, high MAP17 levels might be used to select some patients with other tumors for which bortezomib are not currently indicated, such as breast cancer.

Sarcomas constitute a rare type of mesenchymal tumor with a high rate of mortality in children and young adults [141]. These tumors include approximately 60 different subtypes and more than 50 types of benign tumors [142, 143]. This heterogeneous group of tumors exhibits different MAP17 expression levels, and the expression of this protein was correlated with response to bortezomib in a panel of sarcoma cell lines [42]. In addition, forced expression of MAP17 in AA and AW sarcoma cells with low MAP17 levels makes these cells more sensitive to bortezomib treatment. However, downregulation of MAP17 expression by specific shRNA in sarcoma cell lines with higher MAP17 levels showed a diminished sensitivity to bortezomib treatment [42]. These results are also true for patient-derived xenografts (PDXs), showing that the bortezomib sensitivity of these tumors implanted in mice was based on MAP17 levels [42].

Interestingly, the finding that MAP17 is associated with a variety of inflammatory diseases such as Crohn’s disease, COPD and psoriasis provides an avenue to test bortezomib as an effective inhibitor of these chronic inflammatory diseases.

On the other hand, cells overexpressing MAP17 were shown to be more sensitive to treatment with cisplatin, oxaliplatin or gemcitabine [36]. Additionally, tumor cells overexpressing MAP17 were shown to be more sensitive to radiation, and this sensitivity disappeared with antioxidant treatment [134]. The variable sensitivity of cells to different drugs based on differing MAP17 levels makes it possible to use MAP17 expression levels as a marker of response to therapies inducing oxidative stress. MAP17 expression is associated with an SGLT-dependent ROS increase that acts as a second messenger, enhancing tumorigenesis. While a mild increase in ROS has been shown to activate signaling cascades that upregulate tumorigenic processes, further ROS increases lead to a potentially toxic cellular environment and thus programmed cell death [38]. The hypothesis is that tumors expressing both high levels of ROS and MAP17 proteins can benefit from therapies such as cisplatin or radiotherapy that further increase oxidative stress as this could sensitize them to cell death. In a cohort of patients with larynx cancer, MAP17 expression was associated with overall survival and laryngoesophageal dysfunction-free survival. Locoregional control in patients with high MAP17 showed better outcomes than those with low MAP17. These results are consistent with what others have presented in cervical cancer, in which high levels of MAP17 correlated with improved patient survival after treatment [36] . Furthermore, proof of principle experiments in vitro demonstrated that antioxidant treatments reduced the sensitivity of MAP17-expressing HeLa cells to a value similar to parental cells, confirming the relevance of the oxidative status of the tumors in their response to radiation [36, 134]. In addition, patients with high MAP17 and p-γH2AX, had better prognosis after therapy with cisplatin [135]. Our published data show that high levels of pH2AX correlate with better prognosis after treatment with DNA-damage agents such as cisplatin and radiotherapy, especially if cisplatin is given at optimal doses. These data are suggestive of DNA Damage Response (DDR) pathway activation, perhaps as an indicator of low DNA-repair ability in response to DNA-damaging agents in tumor therapy. The fact that doses of cisplatin are important for survival seems to confirm this hypothesis. In line with this, wild-type P53 with high levels of pH2AX confers a good prognosis [135], suggesting that P53 activity is essential to drive the physiological response of apoptosis (or senescence) to DNA-damage agents in tumors with DDR activated. Therefore, high levels of MAP17 induced ROS that in turn increases DNA-damage and DDR signaling. Upon further DNA-damage and further increase in ROS as induced by cisplatin and radiotherapy treatment, tumors with higher oxidative stress (higher MAP17 and higher ROS as denoted by higher pH2AX) are more suitable to undergo apoptosis in the presence of P53 activity [144].

Finally, phloridzin, an inhibitor of the Na^+^-glucose transporter, or furosemide, an inhibitor of the Na^+^-K^+^-2Cl^−^ symporter that reduces the reabsorption of NaCl and decreases the positive potential derived from K^+^ recycling, have been tested in cells with high MAP17 levels [36, 40, 75]. Phloridzin acts on SGLT1 and SGLT2, and due to the described effect of MAP17 on SGLT2, it has been hypothesized that ROS production due to MAP17 occurs through SGLT2 activation [75]. Previous studies demonstrated that activation of SGLT1 rescued enterocytes from apoptosis by activating PI3K [145] and that inhibition of this membrane transport with phloridzin also inhibited MAP17-dependent ROS increase and cell proliferation [40].

## Conclusions

**Fig. 3:**
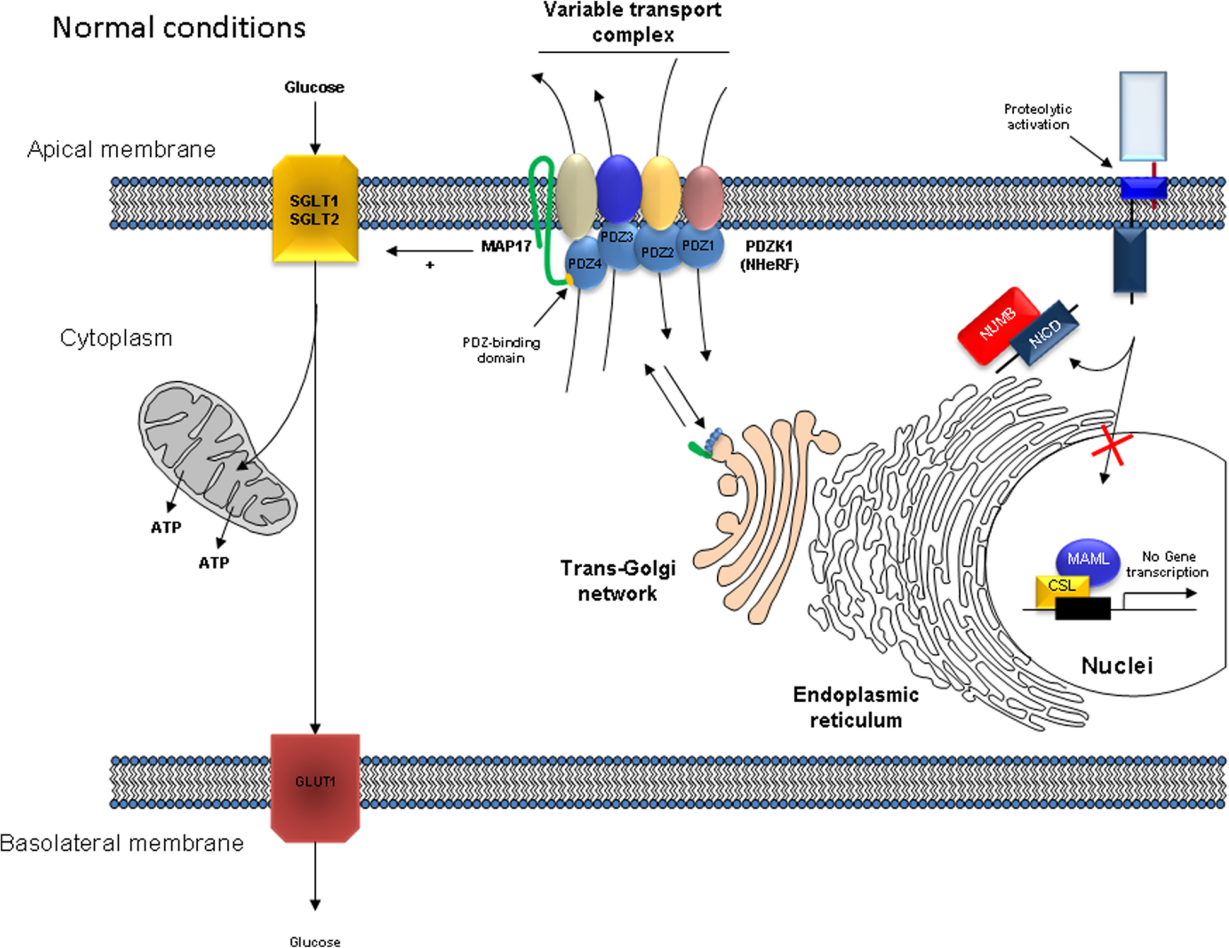
MAP17 function in normal cells. MAP17 interaction with PDZK1 allows the assembly of a multimeric and variable complex responsible for cation/anion transport across the membrane. This complex potentiates the uptake of glucose by SGLT1/2 and the subsequent transport of glucose by GLUT1 from basolateral membrane to the bloodstream. Presence of MAP17-PDZK1 complexes in the trans-Golgi network allows cells to adapt to a change in ion transport requirements. The Notch pathway remains inactivated due to NUMB-NICD interaction, which labels NICD for ubiquitination and degradation

**Fig. 4:**
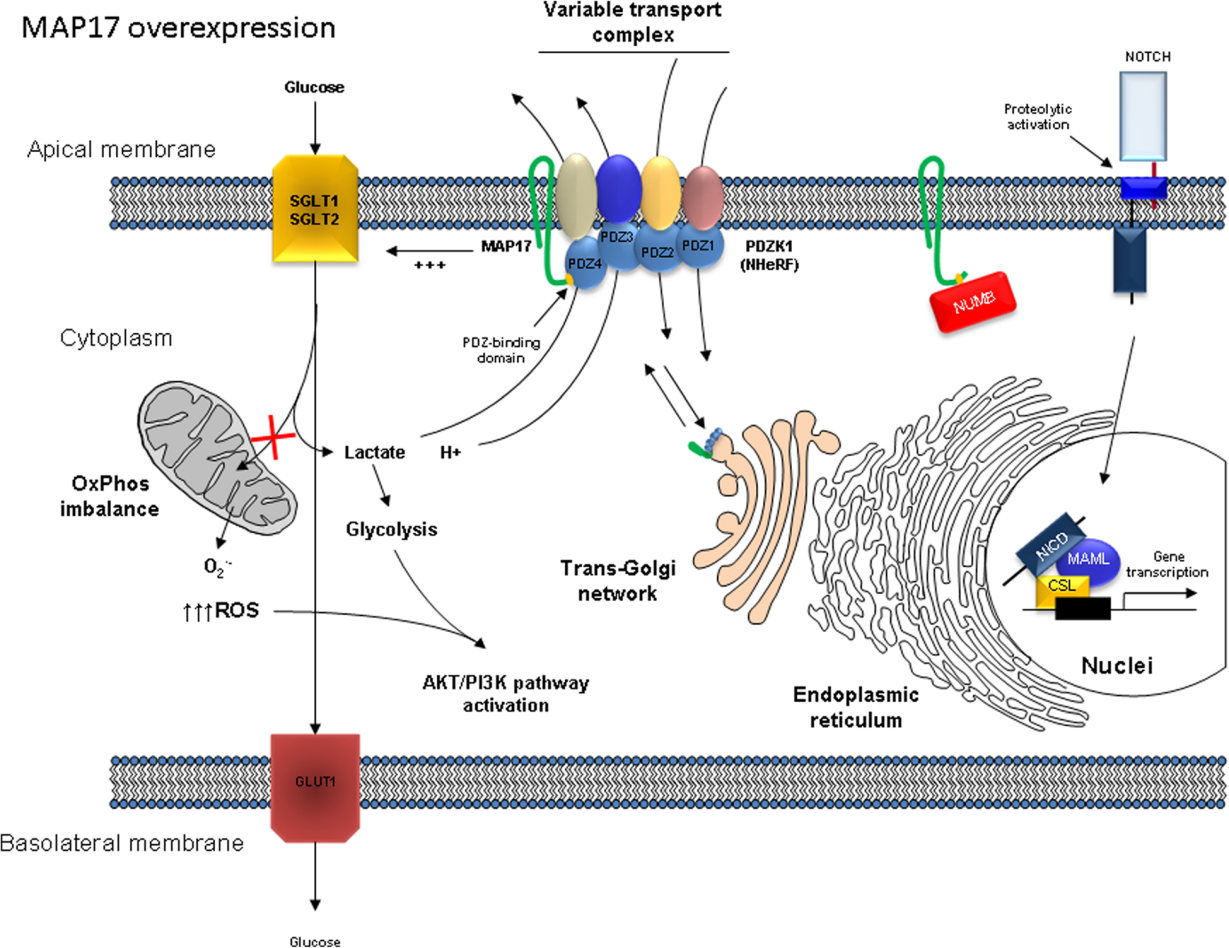
Pathway alterations induced by MAP17 overexpression. MAP17 overexpression causes an increase in glucose uptake and Notch pathway activation. NUMB interaction with MAP17 allows for activation of the Notch pathway, while the increased glucose uptake together with the OXPHOS imbalance drives increased ROS production, changes to glycolytic metabolism, and activation of the AKT/PI3K pathway

All data currently existing about the role of MAP17 allow us to visualize two different regulation scenarios: the first about the role of this protein in normal cells (Fig. 3) and the other showing all the changes that occur due to MAP17 overexpression (Fig. 4). At the latter case, MAP17 is directly responsible for the activation of the Notch pathway due to its described interaction with NUMB and for disruption of glucose homeostasis due to its effects on the SGLT proteins. The interaction of MAP17 with SGLT proteins can increase glycolysis and ROS production, allowing for the activation of the AKT/PI3K signaling pathway.

MAP17 overexpression has been shown to be an important event triggering inflammation and increased tumorigenic properties. Given its roles in modifying signaling pathways and increasing ROS levels, MAP17 represents an important target for the design of specific treatments. However, the lack of an enzymatic activity by MAP17 necessitates the use of treatments that target its interactions, such as those that disrupt MAP17-PDZK1 or MAP17-NUMB binding (Table 1). These strategies could be used to avoid both ROS increase and the activation of stem cell-like genes, which could be especially useful in inflammatory diseases with confirmed high MAP17 levels. However, in tumors, the higher ROS levels due to MAP17 overexpression require treatments that produce a subsequent ROS increase, enough to trigger apoptosis in cancer cells (Table 1). Although the small fraction already known has shown its pleiotropic effects, more research is needed to completely unveil the importance of MAP17 in cell homeostasis.

**Table 1:** Observed effects due to MAP17 high expression in different cell lines and tumor types

Concurring factors	Tumor/ cell type	Effect	Treatment	Ref.
SGLT1 upregulation	Cervical, laryngeal	Better survival	Cisplatin + radiotherapy; Phloridzin, in vitro	[36, 133]
γH2AX phosphorylation	Laryngeal	Better survival	Cisplatin + radiotherapy	[134]
ND	Sarcoma	Poor survival	Cisplatin, oxaliplatin, gemcitabine, radiation	[38, 133]
ND	Breast	N.D.	Bortezomib, in vitro	[31, 36, 39, 43]
ND	Prostate	N.D.	N.D.	[31, 36, 39]
SGLT2 increased activity	–	N.D.	Phloridzin, in vitro	[74]
SLC34A2 downregulation	A549 cells	N.D.	N.D.	[53]
Notch pathway activation through MAP17-NUMB interaction	T47D, HeLa, Calu3, sarcoma cells	N.D.	N.D.	[41]
Reduced NFκB activation	T47D, MDA-MB-231, sarcoma cells	N.D.	Bortezomib, in vitro	[42, 43]
Reduced cell autophagy	T47D, MDA-MB-231 cells	N.D.	Bortezomib, in vitro	[43]
Decreased ERK1/2 phosphorylation	T47D, MDA-MB-231 cells	N.D.	Bortezomib, in vitro	[43]
Inhibition of p21, pRB dephosphorylation	A375, T47D, Me180, HBL100 cells	N.D.	N.D.	[32]
Increased ROS generation	A375, T47D cells	Increased sensitivity	Antioxidants, in vitro	[35, 39, 40]
Activation of AKT-PI3K	RAT1 cells	Increased sensitivity	Antioxidants, in vitro	[33]
p38α phosphorylation	T47D cells, breast tumor	Increased sensitivity	Antioxidants, in vitro	[39]
